# Laparoscopic Surgery for Recurrent Crohn's Disease

**DOI:** 10.1155/2012/381017

**Published:** 2012-01-02

**Authors:** Antonino Spinelli, Matteo Sacchi, Piero Bazzi, Nicoletta Leone, Silvio Danese, Marco Montorsi

**Affiliations:** ^1^Dipartimento e Cattedra di Chirurgia Generale, Istituto Clinico Humanitas IRCCS, Università degli Studi di Milano, Rozzano, Milano, Italy; ^2^IBD Unit, Dipartimento di Gastroenterologia, Istituto Clinico Humanitas IRCCS, Rozzano, Milano, Italy

## Abstract

In spite of the recent improvements in drug therapy, surgery still represents the most frequent treatment for Crohn's disease (CD) complications. Laparoscopy has been widely applied over the last twenty years in colorectal surgery and was associated with lower postoperative pain, shorter hospitalization, faster return to daily activities, and better cosmetic results. Laparoscopy experienced a slower diffusion in inflammatory bowel disease surgery than in oncologic colorectal surgery, but proved to be safe and effective, and is currently considered the gold standard for the treatment of primary uncomplicated ileocolic CD. Indications for laparoscopy in CD have recently been widened to embrace more complicated or recurrent CD. This paper reviews the available data on the subset of recurrent CD patients. The reported results indicate that laparoscopy may be safely applied even in selected recurrent CD cases in hands of IBD surgeons with broad laparoscopic experience.

## 1. Introduction

Crohn's disease (CD) is a chronic and idiopathic inflammation that can affect any part of the gastrointestinal tract. The terminal ileum is the most frequently involved site, and first diagnosis is generally made between the ages of 20 and 30 years. Surgery plays a very important role in the management of CD. 70% to 90% [1] of diagnosed patients eventually require surgery, usually for complications or failure of medical treatment. Approximately 40% to 50% of patients undergoing surgery are likely to need further operations within 10–15 years [2].

Laparoscopic colorectal surgery began in the early 90s and rapidly spread, gaining acceptance for different indications, both benign and malignant.

The reduction of postoperative pain, commonly experienced by laparoscopic patients, allows a faster mobilization and improves pulmonary function [3]; these factors can contribute to lower complications rates [4] and make patients' recovery smoother.

Significantly faster resumption of bowel function, a shorter hospital stay, and a lower overall morbidity are included among the generally mentioned benefits of laparoscopic surgery [5–11]. It is well known that the use of opiate analgesics negatively affects recovery of gastrointestinal function [12]. Laparoscopic approach, probably due to both limited incision extension and bowel manipulation, reduces postoperative pain and morphine administration and leads to rapid resolution of paralytic ileus and discharge from hospital, respectively. Such results were reported also for inflammatory bowel diseases [13].

In particular, CD patients are potentially optimal candidates for laparoscopy because they are mostly young and potentially more concerned about body image and cosmetic results. The high risk of surgical recurrence is a further reason to preserve the integrity of the abdominal wall. Furthermore, laparoscopic surgery might induce less adhesions [14], and since CD patients may undergo repeated surgery during their lives, this means lower risk of surgery for subocclusion. In case of need of subsequent surgery, the resulting operation used to be much easier.

In recurrent CD, the diffusion of laparoscopy was limited by objective technical difficulties and disease-related factors as fragility of inflamed, thickened mesentery and loops, presence of inflammatory masses or abscesses, fistulas, and massive adhesions.

Several studies, including four randomized trials [5, 6, 15, 16] and three meta-analyses [17–19], demonstrated the benefits of laparoscopy for primary small bowel CD regarding short-term outcomes such as postoperative pain, use of medication, complication rates, return to normal bowel habits, hospital stay, and cosmesis. For these reasons, laparoscopy in primary CD is nowadays considered the first choice treatment in most referral surgical centers.

The mean conversion rate reported in the current literature is 11.2% and ranges from 4.8% to 29.2% [17]. The duration of surgery for laparoscopic ileocolic resection can be very similar to open surgery after completion of the learning curve by the surgical team [6, 20, 21].

The safety of laparoscopic ileocolectomy has been proven also in the long-term outcomes by Eshuis and colleagues [22].

Today, surgeons refined their laparoscopic technical skills and got the help of new-generation instruments; indications for CD surgery broadened from uncomplicated ileocaecal resection or simple stoma formation to more complex procedures, even for recurrent disease.

Another major improvement in colorectal surgery has been represented by the introduction of a fast-track perioperative care program, also referred to as enhanced recovery after surgery (*ERAS*) [23, 24], which may reduce hospital stay to 2-3 days after open colorectal surgery [25, 26], even if high readmission rates are reported [25, 27]. Only a few studies evaluated the role of the laparoscopic approach combined to fast-track protocols in enhancing recovery after colorectal surgery and report conflicting results. Basse et al. [28] found no difference between fast-track patients undergone laparoscopic or open resection, while King et al. [29] found a significant reduction of the hospital stay in fast-track patients after laparoscopic surgery. The only randomized, multicentric clinical trial (LAFA study) [30] that investigated both surgical technique (laparoscopic and open) combined with fast-track and standard care demonstrated that the best option is laparoscopic resection embedded in a fast-track care procedure. All studies on laparoscopy and enhanced recovery protocols are focused on colon cancer and have not been validated yet in patients with inflammatory bowel disease, which may have a very different immunologic background.

The few literature reports on recurrent CD treated by laparoscopy will be reviewed in the following section.

## 2. Recurrent Crohn's Disease

In the current literature, there are a few studies investigating the feasibility and safety of laparoscopic resection for recurrent disease [31].

Details on the published studies on the results of laparoscopic surgery for recurrent CD are described in Tables 1 and 2.

Wu et al. [32] compared the results of 46 consecutive patients *who underwent* laparoscopic ileocolic resection for CD with a group of 70 patients treated by conventional open surgery. Within the laparoscopic group, there were 10 recurrent CD patients, 14 complicated CD (abscess/phlegmon) patients, and 22 primary uncomplicated ileocecal CD patients. Surgery in the open group was significantly longer and intraoperative blood loss was higher (both *P* < 0.05). Among the three laparoscopic groups, return of bowel function was similar and shorter than in the open group. Postoperative stay was significantly shorter for laparoscopically treated patients (*P* < 0.01).

Hasegawa et al. [33] reported on 52 patients having undergone 61 laparoscopic procedures for ileal or ileocolonic CD. Sixteen procedures were performed for CD recurrence. The first operation was in about half of the laparoscopic cases. Operative time was significantly longer in the recurrence group (*P* = 0.012).

Moorthy et al. [34] in a series of 57 laparoscopic procedures included 26 laparoscopic operations for CD recurrence, comparing outcomes and conversion rates between operation for primary and for recurrent CD. The conversion rate was 42% (11/26) in the recurrent group and 13% (4/31) in the primary group. The conversion rate was not influenced by the approach (open versus laparoscopic) nor by the type of previous surgery (resection, stoma, drainage, and stricturoplasty). Patients submitted to laparoscopic surgery for recurrent CD experienced a higher conversion rate, but similar outcomes compared to primary CD.

Uchikoshi et al. [35] reported 43 operations for recurrent CD. Surgery started laparoscopically in 23 patients, but 6 were intraoperatively converted to a hand-assisted procedure and 10 to a conventional open surgery, so that in conclusion only 7 patients completed the operation laparoscopically.

Lawes and Motson [36], in a short note, reported 15 laparoscopic procedures on 14 patients for recurrent CD with no conversions. Surgical procedures were not limited to ileocolic resection but included also stricturoplasty, abdominoperineal resection, subtotal colectomy, and small intestine resection. No differences were found in major complication rates and length of stay among patients submitted to surgery for primary or recurrent CD. 

A recently published prospective study from Goyer et al. [37] on 124 attempted ileocolonic resections for CD includes 54 patients with complex disease, defined as recurrent CD after ileocolonic resection (27%) or by the presence of fistula (43%) or abscess (30%). The complex/recurrent CD group was associated with significantly longer operative time (*P* < 0.05), higher conversion rate (*P* < 0.01), and increased performance of temporary stomas (*P* < 0.001), with no difference in postoperative outcome if compared with noncomplicated CD group.

Chaudhray and colleagues [38] described a large series of 59 laparoscopic ileocolic resections (30 for recurrent CD) showing the same benefits observed after primary resection without increased complication rates, delayed discharge, or high conversion rate.

Holubar et al. [39] reported a series of 40 patients undergoing laparoscopy for recurrent ileocolic CD. In 75% of the cases, the resection could be carried out without conversion. Conversion rate was significantly higher than the one reported for primary disease by the same group (25% versus 6.6%; *P* < 0.01). The comparison was made between the completed laparoscopic group (*n* = 30) and the converted group (*n* = 10). The short-term benefits of laparoscopy are reported also in this series of operations for recurrent disease. Among converted patients (25%), the assumption of soft diet (3 versus 4 days; *P* = 0.03) and the length of hospital stay (4 versus 7 days; *P* = 0.002) were significantly delayed.

Broquet et al. [40] in a recent paper compared two groups treated by laparoscopy (29) or conventional surgery (33 procedures in 28 patients) for ileocolonic resection for recurrence of CD; some of these patients were previously treated with open surgery. Conversion rate was high in the laparoscopic group (31% of cases) and was mainly due to intestinal injury, intraoperative discovery of fistula, or difficult intraperitoneal adhesions. In this experience, even when converted, laparoscopy was not reported with higher complication rate than the open group. In conclusion, Broquet et al. recommend laparoscopic approach in selected patients with CD recurrence (previous laparotomies <3, no previous history of peritonitis, nonfistulizing disease).

Bandyopadhyay et al. [41] reported 27 patients treated by laparoscopic ileocolic resection for recurrent CD. The short-term outcome was analogue to the primary laparoscopic ileocolonic resection.

Pinto et al. [42] compared the results of laparoscopic surgery for primary (*n* = 80) and recurrent CD (*n* = 50), not limiting the indication to ileocolic resection, but including also other procedures such as subtotal colectomies. Surgical outcome was similar in both groups. The only statistically significant difference was a longer incision in the group of patients treated for recurrent CD. The incidence of stoma formation was higher in converted cases, even if not statistically significant.

## 3. Conclusions

Laparoscopic surgery represents a widely accepted option for selected CD patients: a broad spectrum of procedures, from simple to very complex, can be technically performed. The main accepted indication remains today ileocecal resection for primary uncomplicated CD.

In summary, even if evidence is lacking and more contributions with larger sample size are needed, the limited experiences available from the literature confirm that the laparoscopic approach to recurrent CD should not be avoided in principle; despite high technical difficulty, in hands of IBD surgeons with a deep expertise in laparoscopic surgery, it can be feasible, safe and lead to significant advantages in the postoperative period. 

Laparoscopy for recurrence will be more often proposable in the near future, due to the increasing number of ileocecal resections already performed by laparoscopy for primary CD.

## Figures and Tables

**Table 1:** Characteristics of studies included in the paper.

Author	Publication year	Type of comparison	Study design	Total procedures/procedures for recurrent CD	Study populations—other details	Stoma	Conversion
Wu et al.	1997	Open (70) versus laparoscopic (46)	Retrospective	116/10	All ileocolic resections; within the laparoscopic group, subgroup analysis for complex, recurrent, and primary uncomplicated CD	NR	11%
Hasegawa et al.	2003	Laparoscopic primary (45) versus recurrent (16)	Retrospective	61/16	All ileocolonic resections; within the laparoscopic group, subgroup analysis for primary operation open or laparoscopic	NR	8.2% (6.7% versus 12.5%)
Uchikoshi et al.	2004	Open (20) versus laparoscopic (23)	Retrospective	43/43	Ileocolic resections and stricturoplasty; subgroup analysis for Lap-assisted and HALS	NR	69.6%**
Moorthy et al.	2004	Laparoscopic primary (31) versus recurrent (26)	Retrospective	57/26	Ileocolic resections, subtotal colectomies; within the laparoscopic group subgroup analysis for converted or not-converted procedures	NR	28% (13% versus 42%)
Lawes and Motson	2006	First versus second versus third laparoscopic approach to CD recurrence	Retrospective	29/29	Ileocolic resections, stricturoplasties, subtotal colectomies, and abdominoperineal resection	NR	0%
Goyer et al.	2009	Patients with complex CD (54) versus patients without complex CD (70)	Prospective	124/54	Ileocolic resections and associated procedures: left colectomy, sigmoid suture, duodenal suture, duodenal suture, unplanned splenectomy, and rectovaginal treatment (open group); cholecystectomy, intestinal resection, right and transverse colectomy and oophorectomy, (lap-group)	39% versus 9%*	37% versus 14%*
Broquet et al.	2010	Open (33) versus laparoscopic (29)	Retrospective	62	Ileocolic resections, stricturoplasties	18% versus 24%	31%
Chaudhray et al.	2010	Laparoscopic primary (29) versus recurrent (30)	Retrospective	59/30	All ileocolic resections	NR	8.5% (10.3% versus 6.7%)
Holubar et al.	2010	Laparoscopic completed (30) versus laparoscopic converted (10)	Retrospective	40	All ileocolic resections	3%	25%
Pinto et al.	2011	Laparoscopic primary (80) versus recurrent (50)	Retrospective	130/50	All ileocolic resections	17% versus 10%	23.8% (18.7% versus 32%)
Bandyopadhyay et al.	2011	No comparison	Retrospective	27	All ileocolic resections	NR	7.4%

NR: not reported.

*P value < 0.05.

**In 6 patients, laparoscopic-assisted reoperation was converted to hand-assisted laparoscopic surgery (HALS).

**Table 2:** Significant short-term (30 days) outcomes.

								Days to		
Authors	Operative time (min)	Loss bood (mL)	Morbidity	Reintervention	Readmission	Mortality	Length of stay (days)	soft diet	flatus	stools
Wu et al.	144 versus 202*	131 versus 245*	10% versus 21%	0 versus 4%	0 versus 4%	0 versus 1%	3.9 versus 7.9*	3-4	2-3	3-4
Hasegawa et al.	180 versus 210	50 versus 80	13% versus 19%	NR	NR	0	8 versus 8	NR	NR	NR
Uchikoshi et al.	204 versus 232	548 versus 361*	25%** versus 18%**	NR	NR	0	42.5 versus 22.4*	32 versus 14*	3.6 versus 2.6*	NR
Hoorthy et al.	127 versus 118	350 versus 273	13.5% versus 15.4%	NR	NR	0	7 versus 8	4 versus 5	NR	NR
Lawes and Motson	100	NR	7%	NR	NR	0	5	NR	NR	NR
Goyer et al.	214 versus 191*	NR	17% versus 17%	0 versus 2%	NR	0	8 versus 7	NR	NR	NR
Broquet et al.	226 versus 215	NR	30% versus 38%	6% versus 7%	NR	0	9 versus 9	NR	NR	NR
Chaudhray et al.	85 versus 125*	NR	24% versus 17%	7% versus 3%	7% versus 3%	0	3 versus 3	NR	NR	NR
Holubar et al.	159 versus 165	100 versus 150	10% versus 30%	0% versus 10%	3% versus 10%	0	4 versus 7*	3 versus 4	3 versus 4	4 versus 4
Pinto et al.	182 versus 201	161 versus 201	36% versus 40%	10% versus 6%	NR	0	6.7 versus 7.4	4 versus 4	NR	4.6 versus 4.3
Bandyopadhyay et al.	110 (70–170)	50 (25–250)	7.4%	NR	0	0	4 (2–7)	1	NR	2 (1–3)

*P value < 0.05.

**Wound infection.

NR: not reported days.

## References

[1] Milsom JW (2005). Laparoscopic surgery in the treatment of Crohn’s disease. *Surgical Clinics of North America*.

[2] Hasegawa H, Watanabe M, Nishibori H, Okabayashi K, Hibi T, Kitajima M (2003). Laparoscopic surgery for recurrent Crohn’s disease. *British Journal of Surgery*.

[3] Schwenk W, Bohm B, Witt C, Junghans T, Grundel K, Muller JM (1999). Pulmonary function following laparoscopic or conventional colorectal resection: a randomized controlled evaluation. *Archives of Surgery*.

[4] Boni L, Benevento A, Rovera F (2006). Infective complications in laparoscopic surgery. *Surgical Infections*.

[5] Bemelman WA, Slors JF, Dunker MS (2000). Laparoscopic-assisted vs open ileocolic resection for Crohn’s disease. A comparative study. *Surgical Endoscopy*.

[6] Benoist S, Panis Y, Beaufour A, Bouhnik Y, Matuchansky C, Valleur P (2003). Laparoscopic ileocecal resection in Crohn’s disease: a case-matched comparison with open resection. *Surgical Endoscopy and Other Interventional Techniques*.

[7] Kirat HT, Pokala N, Vogel JD, Fazio VW, Kiran RP (2010). Can laparoscopic ileocolic resection be performed with comparable safety to open surgery for regional enteritis: data from national surgical quality improvement program. *American Surgeon*.

[8] Duepree HJ, Senagore AJ, Delaney CP, Brady KM, Fazio VW (2002). Advantages of laparoscopic resection for ileocecal Crohn’s disease. *Diseases of the Colon and Rectum*.

[9] Schwenk W, Bohm B, Haase O, Junghans T, Muller JM (1998). Laparoscopic versus conventional colorectal resection: a prospective randomised study of postoperative ileus and early postoperative feeding. *Langenbeck’s Archives of Surgery*.

[10] Msika S, Iannelli A, Deroide G (2001). Can laparoscopy reduce hospital stay in the treatment of Crohn’s disease?. *Diseases of the Colon and Rectum*.

[11] Salimath J, Jones MW, Hunt DL, Lane MK (2007). Comparison of return of bowel function and length of stay in patients undergoing laparoscopic versus open colectomy. *Journal of the Society of Laparoendoscopic Surgeons*.

[12] Luckey A, Livingston E, Tache Y (2003). Mechanisms and treatment of postoperative ileus. *Archives of Surgery*.

[13] Casillas S, Delaney CP (2005). Laparoscopic surgery for inflammatory bowel disease. *Digestive Surgery*.

[14] Zmora O (2003). Laparoscopy for Crohn’s disease. *Seminars in Laparoscopic Surgery*.

[15] Milsom JW, Hammerhofer KA, Bohm B (2001). Prospective, randomized trial comparing laparoscopic vs. conventional surgery for refractory ileocolic Crohn’s disease. *Diseases of the Colon and Rectum*.

[16] Maartense S, Dunker MS, Slors FM (2006). Laparoscopic-assisted versus open ileocolic resection for Crohn’s disease: a randomized trial. *Annals of Surgery*.

[17] Tan JJY, Tjandra JJ (2007). Laparoscopic surgery for Crohn’s disease: a meta-analysis. *Diseases of the Colon and Rectum*.

[18] Rosman AS, Melis M, Fichera A (2005). Metaanalysis of trials comparing laparoscopic and open surgery for Crohn’s disease. *Surgical Endoscopy and Other Interventional Techniques*.

[19] Tilney HS, Constantinides VA, Heriot AG (2006). Comparison of laparoscopic and open ileocecal resection for Crohn’s disease: a metaanalysis. *Surgical Endoscopy and Other Interventional Techniques*.

[20] Tabet J, Hong D, Kim CW, Wong J, Goodacre R, Anvari M (2001). Laparoscopic versus open bowel resection for Crohn’s disease. *Canadian Journal of Gastroenterology*.

[21] Luan XJ, Gross E (2000). Laparoscopic assisted surgery for Crohn’s disease an initial experience and results. *Journal of Tongji Medical University*.

[22] Eshuis EJ, Slors JF, Stokkers PC (2010). Long-term outcomes following laparoscopically assisted versus open ileocolic resection for Crohn’s disease. *British Journal of Surgery*.

[23] Wilmore DW, Kehlet H (2001). Management of patients in fast track surgery. *British Medical Journal*.

[24] Fearon KC, Ljungqvist O, Von Meyenfeldt M (2005). Enhanced recovery after surgery: a consensus review of clinical care for patients undergoing colonic resection. *Clinical Nutrition*.

[25] Basse L, Thorbol JE, Lossl K, Kehlet H (2004). Colonic surgery with accelerated rehabilitation or conventional care. *Diseases of the Colon and Rectum*.

[26] Andersen J, Kehlet H (2005). Fast track open ileo-colic resections for Crohn’s disease. *Colorectal Disease*.

[27] Kariv Y, Delaney CP, Senagore AJ (2007). Clinical outcomes and cost analysis of a “fast track" postoperative care pathway for ileal pouch-anal anastomosis. A case control study. *Diseases of the Colon and Rectum*.

[28] Basse L, Jakobsen DH, Bardram L (2005). Functional recovery after open versus laparoscopic colonic resection: a randomized, blinded study. *Annals of Surgery*.

[29] King PM, Blazeby JM, Ewings P (2006). Randomized clinical trial comparing laparoscopic and open surgery for colorectal cancer within an enhanced recovery programme. *British Journal of Surgery*.

[30] Vlug MS, Wind J, Hollmann MW (2011). Laparoscopy in combination with fast track multimodal management is the best perioperative strategy in patients undergoing colonic surgery: a randomized clinical trial (LAFA-study). *Annals of Surgery*.

[31] Heimann TM, Greenstein AJ, Lewis B, Kaufman D, Heimann DM, Aufses AH (1998). Comparison of primary and reoperative surgery in patients with Crohn’s disease. *Annals of Surgery*.

[32] Wu JS, Birnbaum EH, Kodner IJ, Fry RD, Read TE, Fleshman JW (1997). Laparoscopic-assisted ileocolic resections in patients with Crohn’s disease: are abscesses, phlegmons, or recurrent disease contraindications?. *Surgery*.

[33] Hasegawa H, Watanabe M, Nishibori H, Okabayashi K, Hibi T, Kitajima M (2003). Laparoscopic surgery for recurrent Crohn’s disease. *British Journal of Surgery*.

[34] Moorthy K, Shaul T, Foley RJ (2004). Factors that predict conversion in patients undergoing laparoscopic surgery for Crohn’s disease. *American Journal of Surgery*.

[35] Uchikoshi F, Ito T, Nezu R (2004). Advantages of laparoscope-assisted surgery for recurrent Crohn’s disease. *Surgical Endoscopy and Other Interventional Techniques*.

[36] Lawes DA, Motson RW (2006). Avoidance of laparotomy for recurrent disease is a long-term benefit of laparoscopic resection for Crohn’s disease. *British Journal of Surgery*.

[37] Goyer P, Alves A, Bretagnol F, Bouhnik Y, Valleur P, Panis Y (2009). Impact of complex crohn’s disease on the outcome of laparoscopic ileocecal resection: a comparative clinical study in 124 patients. *Diseases of the Colon and Rectum*.

[38] Chaudhary B, Glancy D, Dixon AR (2011). Laparoscopic surgery for recurrent ileocolic Crohn’s disease is as safe and effective as primary resection. *Colorectal Disease*.

[39] Holubar SD, Dozois EJ, Privitera A (2010). Laparoscopic surgery for recurrent ileocolic Crohn’s disease. *Inflammatory Bowel Diseases*.

[40] Broquet A, Bretagnol F, Soprani A (2010). A laparoscopic approach to iterative ileocolic resection for the recurrence of Crohn’s disease. *Surgical Endoscopy*.

[41] Bandyopadhyay D, Sagar PM, Mirnezami A, Lengyel J, Morrison C, Gatt M (2011). Laparoscopic resection for recurrent Crohn’s disease: safety, feasibility and short-term outcomes. *Colorectal Disease*.

[42] Pinto RA, Shawki S, Narita K (2011). Laparoscopy for recurrent Crohn’s disease: how do the results compare with the results for primary Crohn’s disease?. *Colorectal Disease*.

